# Supplementary material for: Genetic risk factors for severe and fatigue dominant long COVID and commonalities with ME/CFS identified by combinatorial analysis
Source: J Transl Med. 2023 Nov 1;21:775. doi: 10.1186/s12967-023-04588-4 (PMC10621206; doi:10.1186/s12967-023-04588-4)
Supplement: Supplementary file 5 — Additional file 5. Supplementary Material section. [file 12967_2023_4588_MOESM5_ESM.docx]

**Author(s):** Krystyna Taylor, Matthew Pearson, Sayoni Das, Jason Sardell, Karolina Chocian, Steve Gardner

**Date:** 29 September 2023

**Status:** v10

Supplementary Data

Long COVID Manuscript

Contents

[1 Supplementary File List 3](#_Toc146879750)

[2 Supplementary Information 4](#_Toc146879751)

[2.1 Sano Genetics Long COVID GOLD Study 4](#_Toc146879752)

[2.2 Genotype Quality Control 7](#_Toc146879753)

[2.3 Gene Annotation Data Sources 7](#_Toc146879754)

[2.4 Comparison of Long COVID with ME/CFS 8](#_Toc146879755)

[2.5 Expanded Genotype Analysis 9](#_Toc146879756)

[2.6 Genetic Ancestry Analysis of GOLD study 10](#_Toc146879757)

[2.7 PLINK GWAS Analysis 10](#_Toc146879758)

[2.8 Cohort Analysis 11](#_Toc146879759)

[2.9 Combinatorial Analysis 11](#_Toc146879760)

[2.10 Tissue or Cell Expression Profile of Long COVID Genes 12](#_Toc146879761)

[2.11 Cross-Disease Analysis 14](#_Toc146879762)

[2.12 Genes Previously Reported in Long COVID Transcriptomics Analysis 17](#_Toc146879763)

[2.13 Gene Tractability Analysis 18](#_Toc146879764)

[2.14 Gene Disease Associations 20](#_Toc146879765)

[3 References 22](#_Toc146879766)

# Supplementary File List

1. **Supplementary File 1**: Long COVID GOLD Study - Data Dictionary.xlsx

The data dictionary for the Long COVID GOLD Study is available in this supplementary file.

1. **Supplementary File 2**: Long_COVID_studies_critical_snps.xlsx

List of disease-associated critical SNPs and genes identified by the PrecisionLife platform in the three long COVID cohorts. Additional columns include gene associations from publicly available cell/tissue-specific eQTL and chromatin-interaction (Hi-C) data.

1. **Supplementary File 3**: Pathway enrichment of long COVID Disease Signatures.xlsx

Pathway enrichment analysis results for disease signatures identified in Severe and Fatigue Dominant long COVID cohorts. The enrichment analysis was performed using g:Profiler using Gene Ontology, KEGG, Reactome and WikiPathways as data sources and considering only genes with at least one annotation as background genes. Only significant results (*p* < 0.05) after applying multiple testing correction were reported from the enrichment analysis.

1. **Supplementary File 4**: Severe Cohort Disease signatures associated with symptom-based scores.xlsx

List of disease signatures that are correlated with symptom-based scores such as increase in respiratory, fatigue or mental health change scores prior to and after COVID.

# Supplementary Information

## Sano Genetics Long COVID GOLD Study

Supplementary Table 1: Scores generated for respiratory, fatigue and mental health symptoms before and after COVID using participant questionnaire responses in the GOLD study.

| Symptom-based score | Participant reported scores derived from questionnaire responses | Attributes from questionnaire response used to calculate change in symptoms before and after COVID |
| --- | --- | --- |
| Respiratory Change | Change in breathlessness at rest | breathlessness_rest_current - breathlessness_rest_pre_covid |
|  | Change in breathlessness after dressing | breathlessness_dressing_current - breathlessness_dressing_pre_covid |
|  | Change in breathlessness after climbing stars | breathlessness_climbing_stairs_current - breathlessness_climbing_stairs_pre_covid |
| Fatigue Change | Change in mobility problems | mobility_problems_severity_now - mobility_problems_severity_pre_covid |
|  | Change in fatigue | fatigue_now - fatigue_pre_covid |
|  | Change in personal care difficulties | personal_care_difficulties_now - personal_care_difficulties_pre_covid |
|  | Change in difficulties to carry on usual activities | usual_activities_difficulty_now -  usual_activities_difficulty_pre_covid |
|  | Change in muscle pain or discomfort | muscle_pain_now -  muscle_pain_pre_covid |
| Mental Health Change | Change in anxiety | anxiety_now - anxiety_pre_covid |
|  | Change in depression | depression_now - depression_pre_covid |
| Total Change | Total change in Respiratory, Fatigue or Mental Health scores | Respiratory score + Fatigue score + Mental Health score |


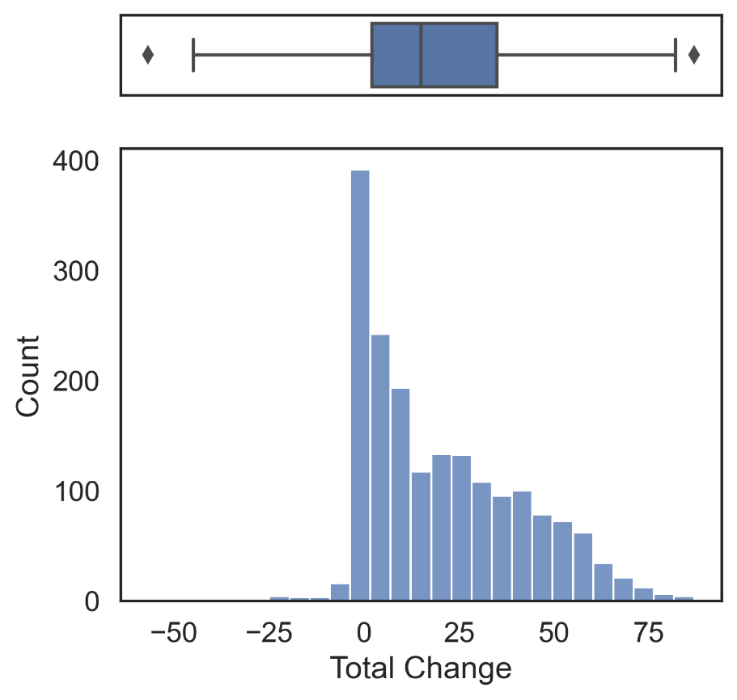


Supplementary Figure 1: Distribution of the ‘Total Change’ score for all individuals in the GOLD cohort.

Supplementary Table 2: List of top ranking (n=35) words in the free-text responses of 1,489 (81.41%) individuals in the GOLD study with a COVID diagnosis (n=1,829) along with Severe and Fatigue Dominant cases to the question on other symptoms individuals in the GOLD long COVID cohort have experienced since their illness that was not covered in the questionnaire. The rank of the words reflects their weights based on their incidence within each group.

| Rank | All GOLD study individuals with COVID diagnosis (*n*=1,828) | | Severe cases  (*n*=459) | | Fatigue Dominant cases  (*n*=477) | |
| --- | --- | --- | --- | --- | --- | --- |
| 1 | **Word** | **Count** | **Word** | **Count** | **Word** | **Count** |
| 2 | smell | 125 | headache | 52 | pain | 62 |
| 3 | headache | 112 | tinnitus | 48 | headache | 57 |
| 4 | pain | 98 | pain | 46 | tinnitus | 53 |
| 5 | tinnitus | 97 | issue | 35 | issues | 37 |
| 6 | taste | 96 | POTS | 30 | symptom | 34 |
| 7 | issue | 74 | smell | 30 | POTS | 33 |
| 8 | problem | 70 | taste | 29 | smell | 33 |
| 9 | symptom | 65 | dizziness | 29 | dizziness | 31 |
| 10 | loss | 60 | symptom | 29 | problem | 31 |
| 11 | dizziness | 51 | problem | 28 | insomnia | 30 |
| 12 | insomnia | 51 | tachycardia | 27 | tachycardia | 30 |
| 13 | POTS | 43 | skin | 26 | eye | 28 |
| 14 | skin | 42 | loss | 26 | skin | 28 |
| 15 | palpitations | 41 | insomnia | 25 | taste | 27 |
| 16 | fatigue | 39 | eye | 24 | loss | 25 |
| 17 | hand | 39 | hand | 18 | hand | 22 |
| 18 | feeling | 36 | palpitations | 17 | palpitations | 19 |
| 19 | severe | 36 | change | 16 | heart | 17 |
| 20 | eye | 35 | tremor | 16 | change | 16 |
| 21 | tachycardia | 34 | severe | 16 | rashes | 16 |
| 22 | still | 33 | sleep | 14 | leg | 16 |
| 23 | month | 33 | heart rate | 14 | migraine | 15 |
| 24 | muscle | 32 | hair loss | 14 | tremor | 15 |
| 25 | sense | 32 | leg | 14 | sleep | 15 |
| 26 | time | 32 | rashes | 13 | severe | 15 |
| 27 | ear | 31 | fatigue | 13 | muscle | 14 |
| 28 | joint pain | 31 | muscle | 13 | hair loss | 14 |
| 29 | hair loss | 31 | brain fog | 13 | sensitivity | 14 |
| 30 | change | 31 | sensitivity | 13 | heart rate | 14 |
| 31 | leg | 29 | migraine | 12 | fatigue | 14 |
| 32 | tingling | 29 | tingling | 11 | ache | 13 |
| 33 | sleep | 29 | feel | 11 | time | 13 |
| 34 | migraine | 29 | light | 11 | tingling | 13 |
| 35 | long | 28 | heart | 11 | brain fog | 13 |


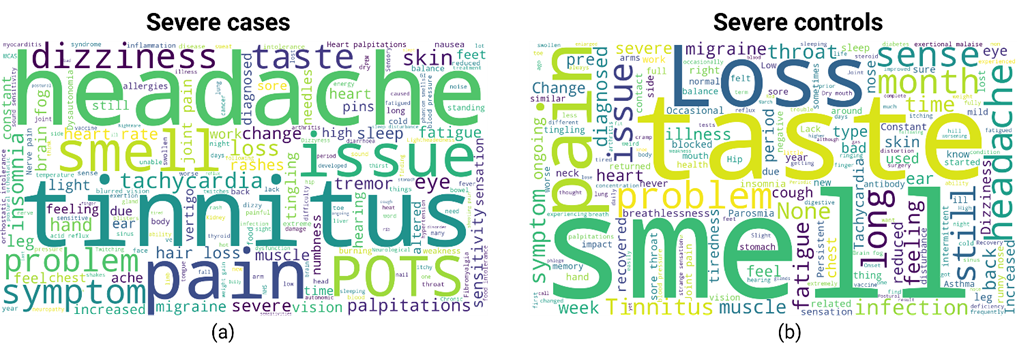


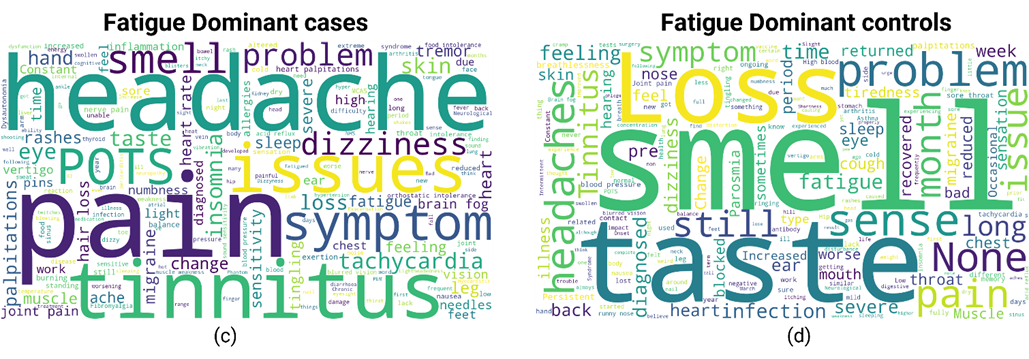


Supplementary Figure 2: Word clouds for (a) Severe cases, (b) Severe controls, (c) Fatigue Dominant cases and (d) Fatigue Dominant controls generated from free-text responses on other symptoms individuals in the GOLD long COVID cohort have experienced since their illness that was not directly covered in the questionnaire. The size of the words in the word clouds indicate the weights based on their incidence within each subcohort.


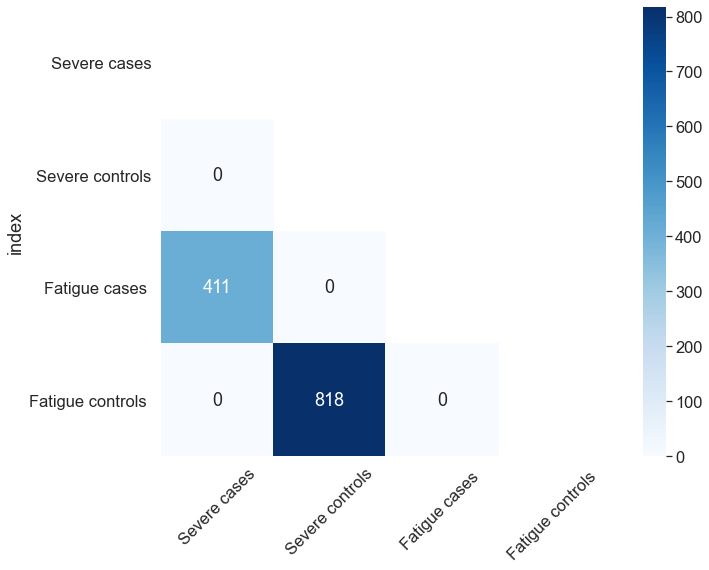


Supplementary Figure 3: Sample overlap between the Severe and Fatigue Dominant long COVID cohorts derived from the GOLD study.


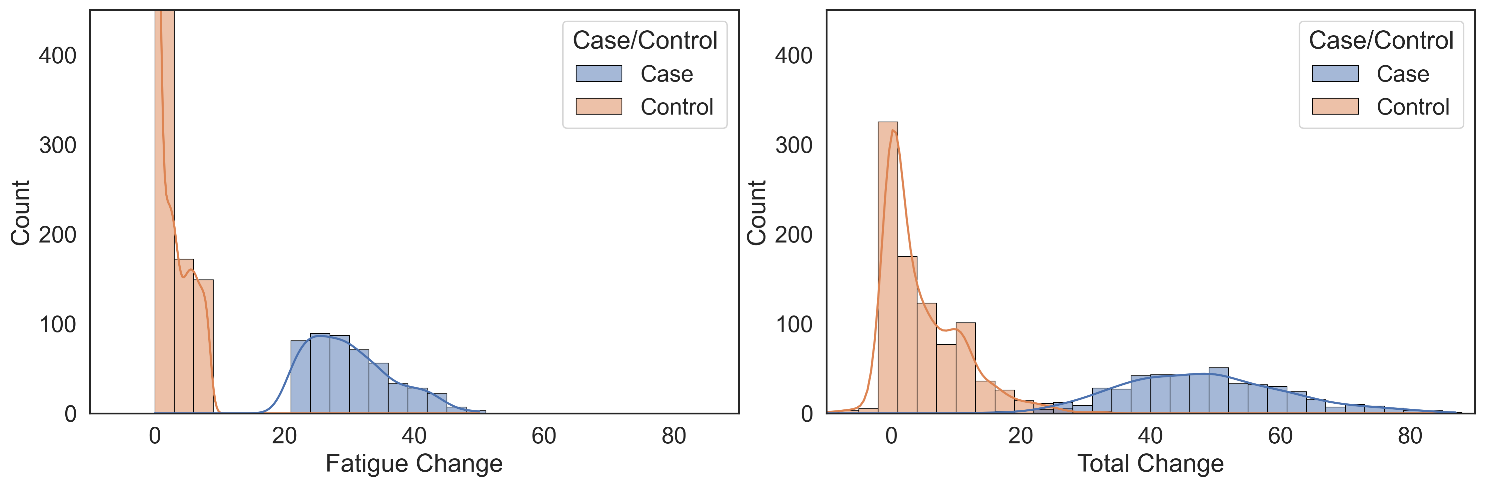


Supplementary Figure 4: Distribution of the ‘Fatigue Change’ and ‘Total Change’ scores for cases and controls in the Fatigue Dominant long COVID cohort.

## Genotype Quality Control

Appropriate quality control of genotype data was performed using GRAF^1^ (Genetic Relationship and Fingerprinting) and PLINK^2^ based on standard quality control procedures to ensure thorough cleaning of the data before it is used for genomic analyses.

This included the following steps:

1. Sample and SNP filtering based on missingness: The filtering for SNPs with missing data (<5%) was followed by filtering of individuals with missing data (<5%) using PLINK.
2. Minor Allele Frequency (MAF) filtering of SNPs: The genotype data would be filtered to exclude SNPs with MAF < 5% using PLINK.
3. Hardy-Weinberg Equilibrium (HWE) filtering: HWE filtering was performed on controls with *p*<10^-10^ using PLINK.
4. Heterozygosity filtering: Samples with extreme (very high or very low) heterozygosity were removed.
5. Sample filtering based on relatedness: GRAF-rel^3^ was used to identify duplicates and closely related subjects in the dataset. After identification of close relatives, only one representative of each closely related family pairs was retained.
6. Sex discrepancy of individuals: Samples that have discrepancies between the sex recorded in the dataset and their sex based on absence/presence of a Y chromosome were removed.

## Gene Annotation Data Sources

Supplementary Table 3: Example public data sources used for annotating genes in the PrecisionLife platform.

| Annotation Type | Data Source(s) |
| --- | --- |
| Genomic Context | Ensembl^4^, Entrez^5^, gnomAD6, HaploReg^7^, ClinVar^8^, Variant Effect Predictor^9^, RegulomeDB^10^ |
| Encoded Protein Structure and Function | Uniprot^11^, InterPro^12^, PDBe-KB^13^ |
| SNP-Disease or Trait Association | dbSNP^14^, GWAS Catalog^15^, PharmGKB^16^, Open Targets Genetics^17^ |
| Gene-Disease or Trait Association | PubMed^18^, PubChem^19^, Open Targets^20^, Mouse Genome Informatics^21^, Human Phenotype Ontology^22^, OMIM^23^ |
| Gene Tissue/Cell Expression | GTEx^24^, Human Protein Atlas^25^, Expression Atlas^26^ |
| Pathways, Interactions or MoA | Reactome^27^, KEGG^28^, Gene Ontology Annotation^29^, PANTHER^30^, WikiPathways^31^, IntAct^32^, STRING^33^, BioGRID^34^, PubMed^18^ |
| Safety / Toxicology | International Mouse Phenotyping Consortium^35^, [Tox21](https://ntp.niehs.nih.gov/whatwestudy/tox21/index.html)^36^, eTox^37^, HeCaToS^38^, Open Targets^20^ |
| Drugs and Chemical Compounds | ChEMBL^39^, DrugBank^40^, PHAROS^41^, PubChem^19^, ProbeMiner^42^, PDBe-KB^13^, ClinicalTrials.gov^43^, Unichem^44^, OpenFDA^45^ |

## Comparison of Long COVID with ME/CFS

Supplementary Table 4: List of 39 SNPs identified in disease signatures associated with long COVID in the Severe and Fatigue Dominant long COVID cohorts that can be linked to 9 genes identified in a combinatorial analysis of UK Biobank ME/CFS patients. 13 SNPs are shown in bold that were identified as critical SNPs in each long COVID cohort.

| Gene | Severe cohort | Fatigue-Dominant cohort |
| --- | --- | --- |
| AKAP1 |  | rs2270278 |
| ATP9A |  | rs1205693 |
| ATP9A |  | rs2147338 |
| ATP9A |  | rs6067922 |
| ATP9A | **rs6096573** | **rs6096573** |
| ATP9A |  | rs6126313 |
| ATP9A | **rs77771672** | **rs77771672** |
| ATP9A | **rs2426361** |  |
| CDON |  | rs4937076 |
| CLOCK |  | **rs62303689** |
| GPC5 | rs1536620 | **rs1536620** |
| GPC5 |  | **rs16946160** |
| GPC5 | rs17267214 | rs17267214 |
| GPC5 | rs2183606 | rs2183606 |
| GPC5 |  | rs35616509 |
| GPC5 | rs6492567 | rs6492567 |
| GPC5 |  | rs7319083 |
| GPC5 |  | rs950725 |
| GPC5 |  | rs9560999 |
| GPC5 |  | rs9589601 |
| GPC5 | rs2149066 |  |
| GPC5 | **rs462954** |  |
| GPC5 | rs59625704 |  |
| GPC5 | **rs9301839** |  |
| GPC5 | rs9523723 |  |
| GPC5 | **rs9560843** |  |
| GPC5 | **rs989236** |  |
| INSR | rs10427021 | rs10427021 |
| INSR | rs6510976 | rs6510976 |
| INSR | rs7317941 | rs7317941 |
| INSR | **rs8110533** | rs8110533 |
| INSR | rs10426094 |  |
| INSR | rs4804103 |  |
| PHACTR2 | rs7742964 | rs7742964 |
| PHACTR2 |  | rs9376793 |
| PHACTR2 |  | rs9496708 |
| PHACTR2 | rs10979 |  |
| SLC15A4 | **rs11059915** | **rs11059915** |
| USP6NL | **rs11257114** |  |

## Expanded Genotype Analysis

Supplementary Table 5: Descriptions of the seven categories that can potentially be assigned to critical SNPs during expanded genotype analyses.

| Category | Observed association of critical SNP minor allele with disease |
| --- | --- |
| Universally causative | Consistently associated with increased odds of disease relative to the homozygous wildtype genotype. |
| Universally protective | Consistently associated with decreased odds of disease relative to the homozygous wildtype genotype. |
| SNP-specific causative effect | Associated with increased odds of disease only when it co-occurs with the minor allele of at least one interacting SNP. Associated with decreased odds of disease in patients who are homozygous for the wildtype alleles of the interacting SNPs. |
| SNP-specific protective effect | Associated with decreased odds of disease only when it co-occurs with the minor allele of at least one SNP. Associated with increased odds of disease in patients who are homozygous for the wildtype alleles of the interacting SNPs. |
| Combination-specific causative effect | Associated with increased odds of disease only when it co-occurs with a specific combination of genotypes at multiple interacting SNPs. Associated with decreased odds of disease in other patients. |
| Combination-specific protective effect | Associated with decreased odds of disease only when it co-occurs with a specific combination of genotypes at multiple interacting SNPs. Associated with increased odds of disease in other patients. |
| Ambiguous | No consistent association with disease risk across expanded genotype signature comparisons. May reflect a highly complex biological interaction, a “false positive” disease signature, or stochastic noise in the data that obscures true biological relationships. |

## Genetic Ancestry Analysis of GOLD study

Supplementary Table 6: Genetic ancestry distribution of samples within the two long COVID cohorts in the GOLD study.

|  | Severe Long COVID *n* = 1,323 | | Fatigue Dominant Long COVID *n* = 1,386 | |
| --- | --- | --- | --- | --- |
|  | **Cases (*n*=459)** | **Controls (*n*=864)** | **Cases  (*n*=477)** | **Controls**  **(*n*=909)** |
| Genetic ancestry [*n*]  S Asian: South Asian  E Asian: East Asian  O Asian: Other Asian  AA: African American | Europe: 426  S Asian: 14  E Asian: 1  African: 1  AA: 1  Hispanic1: 7  Hispanic: 0  O Asian: 1  Other: 8 | Europe: 794  S Asian: 23  E Asian: 9  African: 6  AA: 12  Hispanic1: 5  Hispanic2: 2  O Asian: 9  Other: 3 | Europe: 443  S Asian: 15  E Asian: 0 African: 1  AA: 3  Hispanic1: 6  Hispanic2: 0  O Asian: 1  Other: 8 | Europe: 838  S Asian: 23  E Asian: 10  African: 6  AA: 11  Hispanic1: 7  Hispanic2: 2  O Asian: 9  Other: 3 |

## PLINK GWAS Analysis


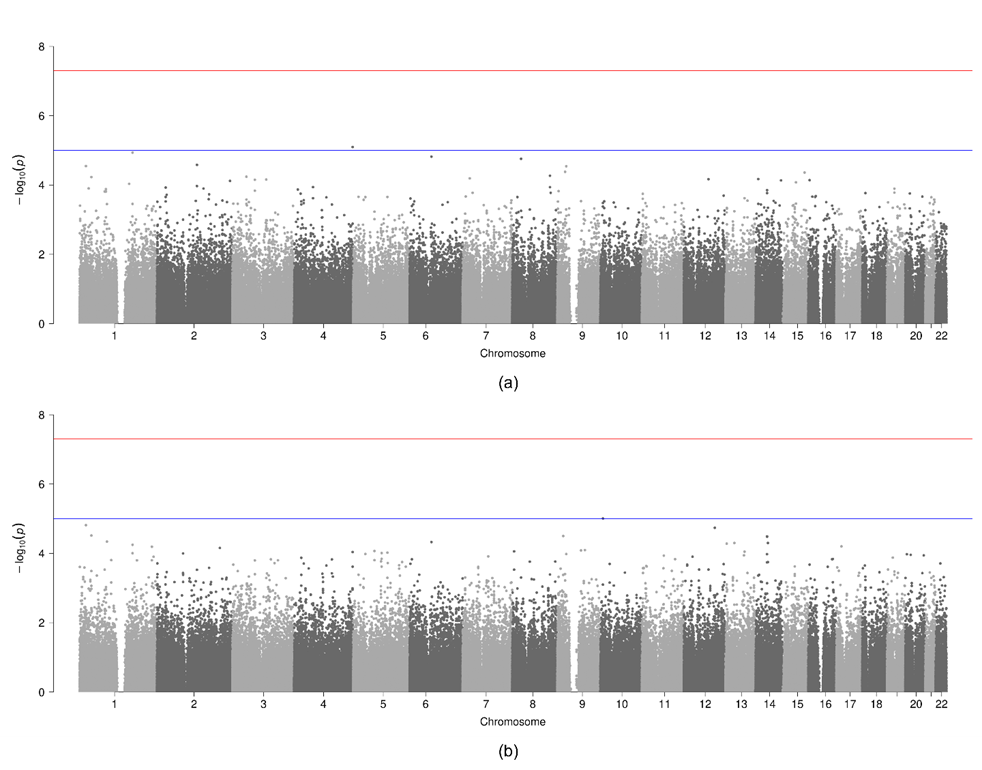
Supplementary Figure 5: Manhattan plots generated using PLINK of genome-wide *p*-values of association for the (a) Severe cohort (*n* = 1,323 where cases = 459 and controls = 864) and (b) Fatigue Dominant cohort (*n* = 1,386 where cases = 477 and controls = 909). The horizontal blue and red lines represent the genome-wide significance values of *p*<1 x 10^-5^ and *p*<5 x 10^-8^ respectively.

## Cohort Analysis


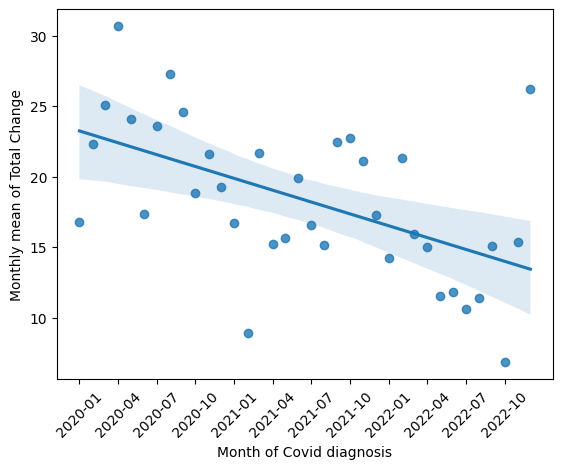


Supplementary Figure 6: Scatter plot showing Total Change with time generated using seaborn package in python. Points in the scatter represent mean total change (across months). To create a regression plot, the dates have been transformed to ordinal numbers (proleptic Gregorian ordinal of the date, where January 1 of year 1 has ordinal 1). The graph shows a regression line and shaded 95% confidence intervals for the line of best fit. This shows a trend of negative correlation of mean total change with time. *p* value = 0.00066 indicates that we can confirm the alternative hypothesis i.e. slope of the regression line is smaller than 0. The correlation coefficient is low (R^2^= 0.2924), which could be caused by the variability of data due to seasonality of the results. Regression and coefficients were calculated using python scipy package (scipy.stats.linregress).

## Combinatorial Analysis

**Supplementary Table 7:** The number of disease signatures (SNP combinations) that pass ancestry confounder analysis and the proportion of non-European cases (based on genetic ancestry) in each long COVID cohort.

| Long COVID Cohort | Disease Signatures that pass ancestry confounder analysis | Non-European Case Count  (% of non-European samples) |
| --- | --- | --- |
| Severe | 1,188 (100%) | 33 (32.35%) |
| Fatigue Dominant | 1,306 (91.0%) | 34 (32.4%) |

## Tissue or Cell Expression Profile of Long COVID Genes


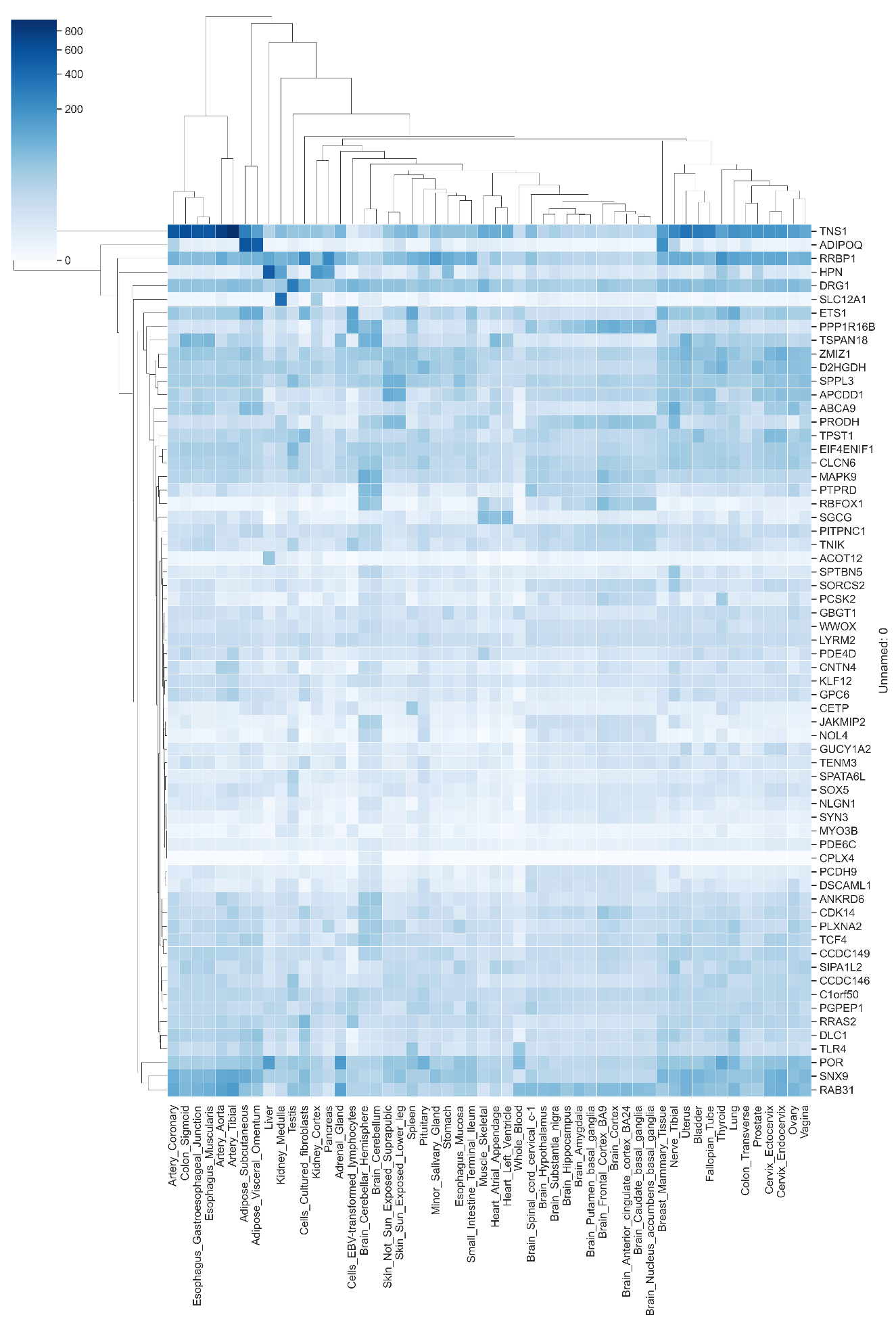


Supplementary Figure 7: Clustered heatmap showing tissue expression profiles from GTEx^24^ for 64 out of 73 long COVID genes identified in the GOLD study with expression information.


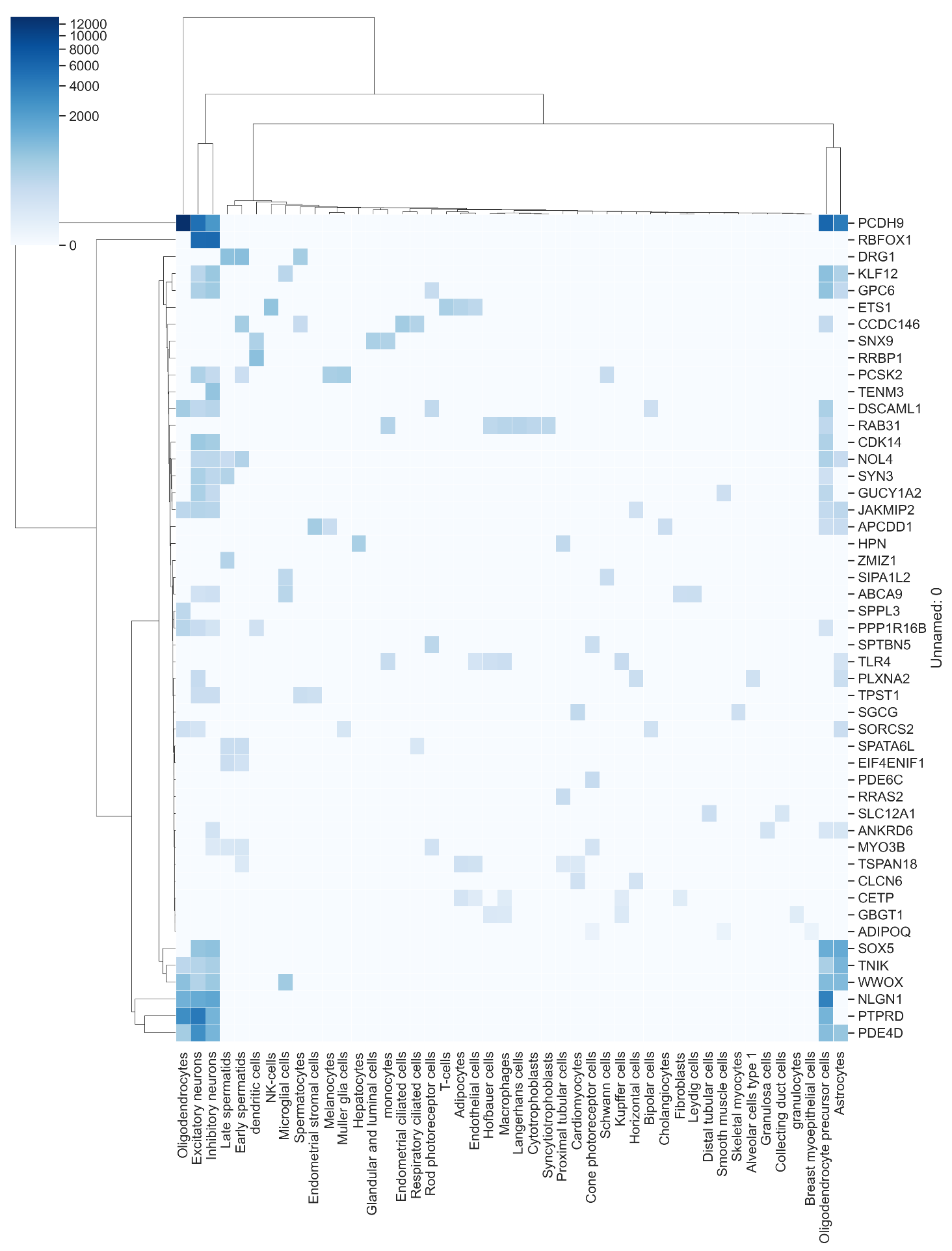


Supplementary Figure 8: Clustered heatmap showing single cell expression profiles from Human Protein Atlas^25^ for 49 out of 73 long COVID genes identified in the GOLD study with expression information.

## Cross-Disease Analysis

**Supplementary Table 8:** Disease-associated genes identified for each indication group that have genetic evidence reported in Open Targets^20^ (version 23.02, released in February 2023). Only genes with target-disease genetic association score > 0.9 out of 1.0 have been used.

| Indication groups | No. of disease-associated genes in indication group (Open Targets target-disease | Overlap with long COVID genes  (*n* = 73) |
| --- | --- | --- |
| Neurodegenerative disease (EFO:0005772) | 170 | 0 |
| Mental or behavioral disease (EFO:0000677) | 97 | 1 |
| Cardiovascular disease (EFO:0000319) | 107 | 0 |
| Gastrointestinal disease (EFO:0010282) | 80 | 0 |
| Autoimmune disease (EFO:0005140) | 40 | 1 |
| Metabolic disease (EFO:0000589) | 410 | 1 |

**Supplementary Table 9:** List of diseases included under each indication group based on Experimental Factor Ontology (EFO) ontology^46^.

| Indication group  (EFO term) | Diseases included under each indication group  (direct descendants in the EFO ontology) |
| --- | --- |
| Neurodegenerative disease (EFO:0005772) | Marchiafava-Bignami Disease  secondary Parkinson disease  Primary progressive aphasia  tauopathy  motor neuron disease  inherited neurodegenerative disorder  cerebellar degeneration  human prion disease  infantile bilateral striatal necrosis  diffuse cerebral and cerebellar atrophy - intractab...  eye degenerative disorder  demyelinating disease  neuroaxonal dystrophy  synucleinopathy |
| Mental or behavioral disease (EFO:0000677) | Atypical progressive supranuclear palsy  Right temporal lobar atrophy  Classical progressive supranuclear palsy  CADASIL  pathological gambling  post-concussion syndrome  internalizing disorder  stress-related disorder  dysphoria  visuospatial impairment  somatoform disorder  gender identity disorder  Sleep Disorder  Kluver-Bucy syndrome  agnosia  anxiety disorder  substance withdrawal syndrome  developmental disorder of mental health  psychosis  occupation-related stress disorder  eating disorder  mood disorder  drug dependence  nervousness  decreased attention  adjustment disorder  alcohol-induced mental disorder  cognitive disorder  drug-induced mental disorder |
| Cardiovascular disease (EFO:0000319) | vascular disease  heart disease  cardiovascular neoplasm  congenital anomaly of cardiovascular system  autoimmune disorder of cardiovascular system |
| Gastrointestinal disease (EFO:0010282) | Genetic digestive tract tumor  Genetic intestinal disease  chemotherapy-induced gastrointestinal mucositis  ventral hernia  stomach rupture  stomach diverticulum  lingual thyroid  immunoproliferative small intestinal disease  Chilaiditi Syndrome  enterocolitis  colon diverticulum  mouth disease  intestinal volvulus  gastric outlet obstruction  gastric antral vascular ectasia  Salivary Gland Acinic Cell Carcinoma  Intestinal Type Adenocarcinoma  Hyperplastic Polyp  Gastric Diffuse Large B-Cell Lymphoma  Duodenal Gastrin-Producing Neuroendocrine Tumor  Digestive System Adenoma  gastrointestinal toxicity  hepatobiliary disease  gastrointestinal ulceration, recurrent, with dysfunctional platelets  small intestine enteropathy  flatulence  stomach disease  pancreas disease  disease of peritoneum  foreign body in gastrointestinal tract  intestinal disease  splenic disease  trichuriasis  staphyloenterotoxemia  paratyphoid fever  oesophagostomiasis  necatoriasis  hymenolepiasis  gastrointestinal tuberculosis  echinostomiasis  dicrocoeliasis  coccidiosis  ascaridiasis  ascariasis  rotavirus infection  upper digestive tract disorder  digestive system neuroendocrine neoplasm  gastrointestinal polyp  neoplasm of oropharynx  gastroesophageal disease  congenital enteropathy due to enteropeptidase deficiency  Cronkhite-Canada syndrome  peptic ulcer disease  digestive system cancer  diarrheal disease  autoimmune disorder of gastrointestinal tract  benign digestive system neoplasm |
| Autoimmune disease (EFO:0005140) | Susac Syndrome  oroficial granulomatosis  malacoplakia  monogenic diabetes  Eosinophilia-Myalgia Syndrome  CNS demyelinating autoimmune di...  STAT3 gain of function  latent autoimmune diabetes in a...  autoimmune bullous skin disease  reactive arthritis  Guillain-Barre syndrome  autoimmune thrombocytopenic pur...  autoimmune thyroid disease  cryoglobulinemia  Autoimmune Hepatitis  Granulomatosis with Polyangiiti...  Myasthenia gravis  anti-neutrophil antibody associ...  Vitiligo  cutaneous lupus erythematosus  Behcet's syndrome  inflammatory bowel disease  antiphospholipid syndrome  juvenile idiopathic arthritis  Sjogren syndrome  rheumatoid arthritis  systemic lupus erythematosus |
| Metabolic disease (EFO:0000589) | Tumor Lysis Syndrome  hyperamylasemia  lipodystrophy  hemochromatosis  acquired metabolic diseas...  lactic acidosis  x-linked warfarin sensiti...  metabolic toxicity  mineral metabolism diseas...  glucose metabolism diseas...  methylmalonic aciduria (c...  hepatic methionine adenos...  hyperprolactinemia  gout  Hypertriglyceridemia  diabetic retinopathy  xanthoma  nutritional disorder  diabetic nephropathy  metabolic syndrome  disorder of organic acid ...  steroid metabolism diseas...  disorder of acid-base bal...  pyrimidine metabolism dis...  purine metabolism disease  porphyrin metabolism dise...  carbohydrate metabolism d...  hyperlipoproteinemia  bilirubin metabolism dise...  disorder of glycosylation  hyperlipidemia  proteostasis deficiencies  inborn errors of metaboli...  steroid dehydrogenase def...  hypoalphalipoproteinemia  developmental anomaly of ...  familial thyroid dyshormo...  chondrocalcinosis  xanthinuria  glutaric aciduria |

## Genes Previously Reported in Long COVID Transcriptomics Analysis

**Supplementary Table 10:** Table showing long COVID genes identified in the GOLD study by the PrecisionLife platform that were reported to be differentially expressed (adjusted *p* value < 0.05) by Thomas et. al^47^ in different cell types (ModelVariant = ‘Serology_AGM’). Upregulated genes have positive log2 fold change (logFC) and downregulated genes have negative logFC.

| Gene | Long COVID cohort | Cell Type | log2 fold change (logFC) | Adjusted  *p* value |
| --- | --- | --- | --- | --- |
| *ABCA9* | Fatigue Dominant | NK cells resting | 6.338 | 0.028 |
| *DRG1* | Fatigue Dominant | T cells CD8 | -0.566 | 0.025 |
| *ETS1* | Severe | T cells CD4 memory resting | 1.796 | 0.000 |
| *ETS1* | Severe | T cells CD8 | -1.224 | 0.031 |
| *ETS1* | Severe | Neutrophils | -1.563 | 0.006 |
| *JAKMIP2* | Fatigue Dominant | T cells CD4 memory resting | 2.142 | 0.048 |
| *JAKMIP2* | Fatigue Dominant | Neutrophils | -2.330 | 0.049 |
| *KLF12* | Fatigue Dominant | T cells CD4 memory resting | 2.072 | 0.000 |
| *KLF12* | Fatigue Dominant | T cells gamma delta | -1.386 | 0.036 |
| *KLF12* | Fatigue Dominant | Neutrophils | -1.519 | 0.025 |
| *LYRM2* | Fatigue Dominant | T cells CD4 memory resting | 0.637 | 0.049 |
| *LYRM2* | Fatigue Dominant | T cells CD8 | -0.840 | 0.021 |
| *LYRM2* | Fatigue Dominant | Neutrophils | -0.842 | 0.016 |
| *LYRM2* | Fatigue Dominant | T cells CD8 | -1.093 | 0.002 |
| *PPP1R16B* | Severe | T cells CD4 memory resting | 1.785 | 0.004 |
| *PPP1R16B* | Severe | Neutrophils | -1.805 | 0.010 |
| *RAB31* | Severe | T cells CD8 | 1.054 | 0.032 |
| *RRAS2* | Severe | T cells CD4 memory resting | 1.730 | 0.010 |
| *RRAS2* | Severe | Neutrophils | -1.725 | 0.025 |
| *RRAS2* | Severe | T cells CD8 | -1.941 | 0.006 |
| *SNX9* | Severe | T cells CD4 memory resting | 1.007 | 0.028 |
| *SPON1* | Fatigue Dominant | Plasma cells | -7.862 | 0.021 |
| *SPPL3* | Fatigue Dominant | T cells CD8 | 1.120 | 0.002 |
| *SPPL3* | Fatigue Dominant | NK cells resting | 1.023 | 0.026 |
| *SPPL3* | Fatigue Dominant | T cells CD8 | 0.944 | 0.016 |
| *SPPL3* | Fatigue Dominant | T cells gamma delta | 0.849 | 0.040 |
| *TCF4* | Severe | B cells memory | 2.066 | 0.046 |
| *TENM3* | Severe | T cells follicular helper | 6.741 | 0.028 |

## Gene Tractability Analysis

**Supplementary Table 11:** Genes identified in the long COVID study with their tractability as drug targets using annotations from OpenTargets^20^ and other annotations from Human Protein Atlas^25^ and PHAROS^41^.

| Gene | Long COVID Cohort | Protein class | PHAROS target classification | Small molecule Tractability | Small molecule Clinical | Antibody Tractability | Drugs | Active Compounds |
| --- | --- | --- | --- | --- | --- | --- | --- | --- |
| *GUCY1A2* | Severe/Fatigue | Enzyme | Tclin | 0.3 | 1 | 0 | 8 | 595 |
| *CETP* | Severe/Fatigue | Transporter | Tchem | 1 | 0.7 | 1 | 4 | 555 |
| *TLR4* | Severe | Enzyme, Transporter | Tchem | 0.3 | 0.7 | 1 | 10 | 506 |
| *TCF4* | Severe/Fatigue | Transcription factor | Tbio | 0 | 0 | 0 | 0 | 334 |
| *MYO3B* | Severe/Fatigue | Enzyme | Tchem | 0.3 | 0 | 0 | 0 | 245 |
| *LARGE1* | Severe/Fatigue |  | Tbio | 0 | 0 | 0 | 0 | 99 |
| *PCSK2* | Severe/Fatigue | Enzyme | Tchem | 0 | 0 | 0.7 | 2 | 8 |
| *PDE6C* | Severe/Fatigue | Enzyme | Tclin | 0.3 | 1 | 0.7 | 2 | 1 |
| *CLCN6* | Severe/Fatigue |  | Tchem | 0 | 0 | 0.7 | 0 | 2 |
| *SOX5* | Severe/Fatigue | Transcription factor | Tbio | 0 | 0 | 0 | 0 | 2 |
| *PRODH* | Severe/Fatigue | Enzyme | Tbio | 0 | 0 | 0 | 2 | 0 |
| *RRAS2* | Severe/Fatigue | RAS pathway related protein | Tbio | 0.7 | 0 | 0.7 | 0 | 0 |
| *ADIPOQ* | Severe | Transporter | Tbio | 0 | 0 | 1 | 0 | 0 |
| *ADGRA3* | Severe/Fatigue | G-protein coupled receptor | Tbio | 0 | 0 | 0 | 0 | 1 |
| *SNX9* | Severe/Fatigue |  | Tbio | 0.7 | 0 | 0.3 | 0 | 0 |
| *PPP1R16B* | Severe/Fatigue |  | Tbio | 0 | 0 | 1 | 0 | 0 |
| *DSCAML1* | Severe |  | Tbio | 0 | 0 | 1 | 0 | 0 |
| *SORCS2* | Severe/Fatigue |  | Tbio | 0 | 0 | 0.7 | 0 | 0 |
| *APCDD1* | Severe/Fatigue |  | Tbio | 0 | 0 | 0.7 | 0 | 0 |
| *ETS1* | Severe | Transcription factor | Tbio | 0.7 | 0 | 0 | 0 | 0 |
| *SGCG* | Severe/Fatigue |  | Tbio | 0 | 0 | 0.3 | 0 | 0 |
| *GPC6* | Severe/Fatigue |  | Tbio | 0 | 0 | 0.3 | 0 | 0 |
| *RAB31* | Severe/Fatigue |  | Tbio | 0 | 0 | 0.3 | 0 | 0 |
| *MAPK9* | Fatigue/Severe | Enzyme | Tchem | 1 | 0.7 | 0 | 6 | 877 |
| *TNIK* | Fatigue/Severe | Enzyme | Tchem | 1 | 0 | 0 | 1 | 765 |
| *CDK14* | Fatigue/Severe | Enzyme | Tchem | 0.3 | 0.7 | 0.3 | 0 | 258 |
| *HPN* | Fatigue/Severe | Enzyme | Tchem | 0 | 0 | 1 | 4 | 179 |
| *POR* | Fatigue | Enzyme | Tbio | 0.3 | 0 | 0 | 13 | 30 |
| *PDE4D* | Fatigue/Severe | Enzyme | Tclin | 1 | 1 | 1 | 26 | 4 |
| *SLC12A1* | Fatigue/Severe | Transporter | Tclin | 0.3 | 1 | 0.3 | 19 | 0 |
| *DRG1* | Fatigue/Severe |  | Tbio | 0 | 0 | 0 | 0 | 4 |
| *NLGN1* | Fatigue/Severe | Transporter | Tbio | 0.3 | 0 | 1 | 0 | 0 |
| *RRBP1* | Fatigue |  | Tbio | 0 | 0 | 0 | 1 | 0 |
| *TNS1* | Fatigue |  | Tbio | 0 | 0 | 0.7 | 0 | 0 |
| *ACOT12* | Fatigue | Enzyme, Transporter | Tbio | 0.7 | 0 | 0 | 0 | 0 |
| *SPON1* | Fatigue/Severe |  | Tbio | 0.7 | 0 | 0 | 0 | 0 |
| *SYN3* | Fatigue/Severe |  | Tbio | 0.7 | 0 | 0 | 0 | 0 |
| *PLXNA2* | Fatigue/Severe |  | Tbio | 0 | 0 | 0.3 | 0 | 0 |
| *CNTN4* | Fatigue/Severe |  | Tbio | 0 | 0 | 0.3 | 0 | 0 |
| *PTPRD* | Fatigue/Severe | Enzyme | Tbio | 0 | 0 | 0.3 | 0 | 0 |
| *SPPL3* | Fatigue/Severe | Enzyme | Tbio | 0 | 0 | 0.3 | 0 | 0 |
| *WWOX* | Fatigue/Severe |  | Tbio | 0 | 0 | 0.3 | 0 | 0 |

## Gene Disease Associations

**Supplementary Table 12:** Genes with prior association to type 2 diabetes in OpenTargets^Error! Reference source not found.^ database.

| Long COVID Cohort | Gene | Known association to type 2 Diabetes (OpenTargets) |
| --- | --- | --- |
| Severe | ***ADIPOQ*** | Scientific literature, Animal models |
| Severe | ***ZMIZ1*** | Genetic association |
| Severe | ***PDE6C*** | Drugs approved and/or in clinical development for indication |
| Severe | ***SGCG*** | Genetic association |
| Severe | ***RAB31*** | Genetic association |
| Severe | ***NOL4*** | Genetic association |
| Severe | ***ETS1*** | Genetic association |
| Fatigue Dominant | ***TNS1*** | Genetic association |
| Fatigue Dominant | ***NLGN1*** | Genetic association |
| Fatigue Dominant | ***PITPNC1*** | Genetic association |
| Fatigue Dominant | ***EIF4ENIF1*** | Genetic association |

Supplementary Table 13: 19 pathways that are significantly enriched in the 73 long COVID genes that were also significantly enriched in at least one of the following indication groups - neurodegenerative, mental or behavioral, cardiovascular, gastrointestinal, autoimmune and metabolic disorders.

| GO Biological process | Long COVID  (*p* value) | Autoimmune disease  (*p* value) | Cardiovascular disease  (*p* value) | Gastrointestinal disease  (*p* value) | Mental or behavioural disease  (*p* value) | Metabolic disease  (*p* value) | Neurodegenerative disease (*p* value) |
| --- | --- | --- | --- | --- | --- | --- | --- |
| anatomical structure development | 0.022 | 0.001673 | 1.01E-14 | 0.00237 | 3.13E-17 | 6.84E-06 | 1.05E-09 |
| cell adhesion | 0.026 | 2.60E-05 | 0.000181 |  |  |  |  |
| cell communication | 0.039 | 1.76E-06 | 8.95E-07 | 0.032485 | 0.000232 |  | 0.009076 |
| cell differentiation | 0.008 | 0.000395 | 1.39E-09 | 0.004574 | 1.71E-13 |  | 3.31E-06 |
| cell-cell signaling | 0.021 | 0.594388 | 9.09E-05 | 0.001775 | 1.77E-06 |  | 0.000316 |
| chemical synaptic transmission | 0.048 |  | 0.037426 |  | 1.03E-06 |  | 3.88E-06 |
| dendrite self-avoidance | 0.045 |  |  |  |  |  |  |
| filopodium assembly | 0.045 |  |  |  |  |  |  |
| foam cell differentiation | 0.014 |  |  |  |  | 0.02689 |  |
| glial cell migration | 0.033 |  |  |  | 0.001138 |  |  |
| modulation of chemical synaptic transmission | 0.008 |  | 0.003983 |  | 2.40E-08 |  | 0.004787 |
| nervous system development | 0.008 |  | 5.16E-05 | 0.023153 | 2.17E-22 | 9.30E-06 | 1.88E-18 |
| neurogenesis | 0.008 |  | 0.000267 | 0.036673 | 7.46E-18 |  | 2.72E-12 |
| neuron cell-cell adhesion | 0.042 |  |  |  |  |  |  |
| positive regulation of cell differentiation | 0.034 | 0.000192 | 0.000622 | 0.0085 | 1.75E-05 |  |  |
| positive regulation of hormone metabolic process | 0.039 |  |  |  |  |  |  |
| positive regulation of protein dephosphorylation | 0.027 |  |  |  |  |  |  |
| presynapse assembly | 0.028 |  |  |  |  |  |  |
| regulation of biological quality | 0.004 |  | 6.66E-12 | 0.000111 | 1.41E-05 | 1.41E-06 | 0.000444 |
| regulation of cell communication | 0.006 | 2.31E-05 | 3.25E-06 | 6.50E-05 | 1.24E-05 |  |  |
| regulation of cellular component biogenesis | 0.028 |  | 0.000692 |  | 0.007012 |  | 0.000388 |
| regulation of signaling | 0.006 | 2.28E-05 | 2.07E-05 | 6.17E-05 | 1.18E-05 |  |  |
| regulation of trans-synaptic signaling | 0.008 |  | 0.004034 |  | 2.44E-08 |  | 0.004861 |
| response to epinephrine | 0.039 |  | 0.010242 |  |  |  |  |
| response to stimulus | 0.029 | 2.53E-06 | 3.65E-08 | 0.04138 | 8.65E-07 | 0.011387 | 0.01398 |
| synaptic membrane adhesion | 0.01 |  |  |  |  |  |  |
| synaptic vesicle clustering | 0.045 |  |  |  |  |  |  |

# References

1. Jin, Y., Schaffer, A.A., Feolo, M., Holmes, J.B. and Kattman, B.L., 2019. GRAF-pop: a fast distance-based method to infer subject ancestry from multiple genotype datasets without principal components analysis. G3: *Genes, Genomes, Genetics*, 9(8), pp.2447-2461.
2. Purcell, S., Neale, B., Todd-Brown, K., Thomas, L., Ferreira, M.A., Bender, D., Maller, J., Sklar, P., De Bakker, P.I., Daly, M.J. and Sham, P.C., 2007. PLINK: a tool set for whole-genome association and population-based linkage analyses. *The American journal of human genetics*, 81(3), pp.559-575.
3. Jin, Y., Schäffer, A.A., Sherry, S.T. and Feolo, M., 2017. Quickly identifying identical and closely related subjects in large databases using genotype data. *PLoS One*, 12(6), p.e0179106.
4. Martin, F.J., Amode, M.R., Aneja, A., Austine-Orimoloye, O., Azov, A.G., Barnes, I., Becker, A., Bennett, R., Berry, A., Bhai, J. and Bhurji, S.K., 2023. Ensembl 2023. Nucleic Acids Research, 51(D1), pp.D933-D941.
5. Brown, G.R., Hem, V., Katz, K.S., Ovetsky, M., Wallin, C., Ermolaeva, O., Tolstoy, I., Tatusova, T., Pruitt, K.D., Maglott, D.R. and Murphy, T.D., 2015. Gene: a gene-centered information resource at NCBI. Nucleic acids research, 43(D1), pp.D36-D42.
6. Koch, L., 2020. Exploring human genomic diversity with gnomAD. Nature Reviews Genetics, 21(8), pp.448-448.
7. Ward, L.D. and Kellis, M., 2016. HaploReg v4: systematic mining of putative causal variants, cell types, regulators and target genes for human complex traits and disease. Nucleic acids research, 44(D1), pp.D877-D881.
8. Landrum, M.J., Chitipiralla, S., Brown, G.R., Chen, C., Gu, B., Hart, J., Hoffman, D., Jang, W., Kaur, K., Liu, C. and Lyoshin, V., 2020. ClinVar: improvements to accessing data. Nucleic acids research, 48(D1), pp.D835-D844.
9. McLaren, W., Gil, L., Hunt, S.E., Riat, H.S., Ritchie, G.R., Thormann, A., Flicek, P. and Cunningham, F., 2016. The ensembl variant effect predictor. Genome biology, 17(1), pp.1-14.
10. Boyle, A.P., Hong, E.L., Hariharan, M., Cheng, Y., Schaub, M.A., Kasowski, M., Karczewski, K.J., Park, J., Hitz, B.C., Weng, S. and Cherry, J.M., 2012. Annotation of functional variation in personal genomes using RegulomeDB. Genome research, 22(9), pp.1790-1797.
11. UniProt Consortium, 2019. UniProt: a worldwide hub of protein knowledge. Nucleic acids research, 47(D1), pp.D506-D515.
12. Paysan-Lafosse, T., Blum, M., Chuguransky, S., Grego, T., Pinto, B.L., Salazar, G.A., Bileschi, M.L., Bork, P., Bridge, A., Colwell, L. and Gough, J., 2023. InterPro in 2022. Nucleic Acids Research, 51(D1), pp.D418-D427.
13. Varadi, M., Berrisford, J., Deshpande, M., Nair, S.S., Gutmanas, A., Armstrong, D., Pravda, L., Al-Lazikani, B., Anyango, S., Barton, G.J. and Berka, K., 2020. PDBe-KB: a community-driven resource for structural and functional annotations. Nucleic Acids Research, 48(D1), pp.D344-D353.
14. Bhagwat, M., 2010. Searching NCBI's dbSNP database. Current protocols in bioinformatics, 32(1), pp.1-19.
15. Buniello, A., MacArthur, J.A.L., Cerezo, M., Harris, L.W., Hayhurst, J., Malangone, C., McMahon, A., Morales, J., Mountjoy, E., Sollis, E. and Suveges, D., 2019. The NHGRI-EBI GWAS Catalog of published genome-wide association studies, targeted arrays and summary statistics 2019. Nucleic acids research, 47(D1), pp.D1005-D1012.
16. Barbarino, J.M., Whirl‐Carrillo, M., Altman, R.B. and Klein, T.E., 2018. PharmGKB: a worldwide resource for pharmacogenomic information. Wiley Interdisciplinary Reviews: Systems Biology and Medicine, 10(4), p.e1417.
17. Ghoussaini, M., Mountjoy, E., Carmona, M., Peat, G., Schmidt, E.M., Hercules, A., Fumis, L., Miranda, A., Carvalho-Silva, D., Buniello, A. and Burdett, T., 2021. Open Targets Genetics: systematic identification of trait-associated genes using large-scale genetics and functional genomics. Nucleic acids research, 49(D1), pp.D1311-D1320.
18. Roberts, R.J., 2001. PubMed Central: The GenBank of the published literature. Proceedings of the National Academy of Sciences, 98(2), pp.381-382.
19. Kim, S., Thiessen, P.A., Cheng, T., Yu, B., Shoemaker, B.A., Wang, J., Bolton, E.E., Wang, Y. and Bryant, S.H., 2016. Literature information in PubChem: associations between PubChem records and scientific articles. Journal of cheminformatics, 8, pp.1-15.
20. Ochoa, D., Hercules, A., Carmona, M., Suveges, D., Gonzalez-Uriarte, A., Malangone, C., Miranda, A., Fumis, L., Carvalho-Silva, D., Spitzer, M. and Baker, J., 2021. Open Targets Platform: supporting systematic drug–target identification and prioritisation. Nucleic acids research, 49(D1), pp.D1302-D1310.
21. Eppig, J.T., Smith, C.L., Blake, J.A., Ringwald, M., Kadin, J.A., Richardson, J.E. and Bult, C.J., 2017. Mouse Genome Informatics (MGI): resources for mining mouse genetic, genomic, and biological data in support of primary and translational research. Systems Genetics: Methods and Protocols, pp.47-73.
22. Köhler, S., Gargano, M., Matentzoglu, N., Carmody, L.C., Lewis-Smith, D., Vasilevsky, N.A., Danis, D., Balagura, G., Baynam, G., Brower, A.M. and Callahan, T.J., 2021. The human phenotype ontology in 2021. Nucleic acids research, 49(D1), pp.D1207-D1217.
23. Amberger, J.S., Bocchini, C.A., Schiettecatte, F., Scott, A.F. and Hamosh, A., 2015. OMIM. org: Online Mendelian Inheritance in Man (OMIM®), an online catalog of human genes and genetic disorders. Nucleic acids research, 43(D1), pp.D789-D798.
24. GTEx Consortium, 2020. The GTEx Consortium atlas of genetic regulatory effects across human tissues. Science, 369(6509), pp.1318-1330.
25. Digre, A. and Lindskog, C., 2021. The human protein atlas—spatial localization of the human proteome in health and disease. *Protein Science,* 30(1), pp.218-233.
26. Papatheodorou, I., Fonseca, N.A., Keays, M., Tang, Y.A., Barrera, E., Bazant, W., Burke, M., Füllgrabe, A., Fuentes, A.M.P., George, N. and Huerta, L., 2018. Expression Atlas: gene and protein expression across multiple studies and organisms. Nucleic acids research, 46(D1), pp.D246-D251.
27. Jassal, B., Matthews, L., Viteri, G., Gong, C., Lorente, P., Fabregat, A., Sidiropoulos, K., Cook, J., Gillespie, M., Haw, R. and Loney, F., 2020. The reactome pathway knowledgebase. Nucleic acids research, 48(D1), pp.D498-D503.
28. Kanehisa, M., Araki, M., Goto, S., Hattori, M., Hirakawa, M., Itoh, M., Katayama, T., Kawashima, S., Okuda, S., Tokimatsu, T. and Yamanishi, Y., 2007. KEGG for linking genomes to life and the environment. Nucleic acids research, 36(suppl_1), pp.D480-D484.
29. Huntley, R.P., Sawford, T., Mutowo-Meullenet, P., Shypitsyna, A., Bonilla, C., Martin, M.J. and O'Donovan, C., 2015. The GOA database: gene ontology annotation updates for 2015. Nucleic acids research, 43(D1), pp.D1057-D1063.
30. Mi, H., Muruganujan, A., Ebert, D., Huang, X. and Thomas, P.D., 2019. PANTHER version 14: more genomes, a new PANTHER GO-slim and improvements in enrichment analysis tools. Nucleic acids research, 47(D1), pp.D419-D426.
31. Martens, M., Ammar, A., Riutta, A., Waagmeester, A., Slenter, D.N., Hanspers, K., A. Miller, R., Digles, D., Lopes, E.N., Ehrhart, F. and Dupuis, L.J., 2021. WikiPathways: connecting communities. Nucleic acids research, 49(D1), pp.D613-D621.
32. Kerrien, S., Aranda, B., Breuza, L., Bridge, A., Broackes-Carter, F., Chen, C., Duesbury, M., Dumousseau, M., Feuermann, M., Hinz, U. and Jandrasits, C., 2012. The IntAct molecular interaction database in 2012. Nucleic acids research, 40(D1), pp.D841-D846.
33. Szklarczyk, D., Gable, A.L., Nastou, K.C., Lyon, D., Kirsch, R., Pyysalo, S., Doncheva, N.T., Legeay, M., Fang, T., Bork, P. and Jensen, L.J., 2021. The STRING database in 2021: customizable protein–protein networks, and functional characterization of user-uploaded gene/measurement sets. Nucleic acids research, 49(D1), pp.D605-D612.
34. Oughtred, R., Rust, J., Chang, C., Breitkreutz, B.J., Stark, C., Willems, A., Boucher, L., Leung, G., Kolas, N., Zhang, F. and Dolma, S., 2021. The BioGRID database: A comprehensive biomedical resource of curated protein, genetic, and chemical interactions. Protein Science, 30(1), pp.187-200.
35. Muñoz-Fuentes, V., Cacheiro, P., Meehan, T.F., Aguilar-Pimentel, J.A., Brown, S.D., Flenniken, A.M., Flicek, P., Galli, A., Mashhadi, H.H., Hrabě de Angelis, M. and Kim, J.K., 2018. The International Mouse Phenotyping Consortium (IMPC): a functional catalogue of the mammalian genome that informs conservation. Conservation genetics, 19, pp.995-1005.
36. Richard, A.M., Huang, R., Waidyanatha, S., Shinn, P., Collins, B.J., Thillainadarajah, I., Grulke, C.M., Williams, A.J., Lougee, R.R., Judson, R.S. and Houck, K.A., 2020. The Tox21 10K compound library: collaborative chemistry advancing toxicology. Chemical Research in Toxicology, 34(2), pp.189-216.
37. Cases, M., Briggs, K., Steger-Hartmann, T., Pognan, F., Marc, P., Kleinöder, T., Schwab, C.H., Pastor, M., Wichard, J. and Sanz, F., 2014. The eTOX data-sharing project to advance in silico drug-induced toxicity prediction. International journal of molecular sciences, 15(11), pp.21136-21154.
38. Verheijen, M., Sarkans, U., Wolski, W., Jennen, D., Caiment, F. and Kleinjans, J., 2022. Multi-omics HeCaToS dataset of repeated dose toxicity for cardiotoxic & hepatotoxic compounds. Scientific data, 9(1), p.699.
39. Gaulton, A., Bellis, L.J., Bento, A.P., Chambers, J., Davies, M., Hersey, A., Light, Y., McGlinchey, S., Michalovich, D., Al-Lazikani, B. and Overington, J.P., 2012. ChEMBL: a large-scale bioactivity database for drug discovery. Nucleic acids research, 40(D1), pp.D1100-D1107.
40. Wishart, D.S., Feunang, Y.D., Guo, A.C., Lo, E.J., Marcu, A., Grant, J.R., Sajed, T., Johnson, D., Li, C., Sayeeda, Z. and Assempour, N., 2018. DrugBank 5.0: a major update to the DrugBank database for 2018. Nucleic acids research, 46(D1), pp.D1074-D1082.
41. Kelleher, K.J., Sheils, T.K., Mathias, S.L., Yang, J.J., Metzger, V.T., Siramshetty, V.B., Nguyen, D.T., Jensen, L.J., Vidović, D., Schürer, S.C. and Holmes, J., 2023. Pharos 2023: an integrated resource for the understudied human proteome. *Nucleic Acids Research*, 51(D1), pp.D1405-D1416.
42. Antolin, A.A., Tym, J.E., Komianou, A., Collins, I., Workman, P. and Al-Lazikani, B., 2018. Objective, quantitative, data-driven assessment of chemical probes. Cell chemical biology, 25(2), pp.194-205.
43. Anand, V., Cahan, A. and Ghosh, S., 2017. Clinical Trials. Gov: A Topical Analyses. AMIA Summits on Translational Science Proceedings, 2017, p.37.
44. Chambers, J., Davies, M., Gaulton, A., Hersey, A., Velankar, S., Petryszak, R., Hastings, J., Bellis, L., McGlinchey, S. and Overington, J.P., 2013. UniChem: a unified chemical structure cross-referencing and identifier tracking system. Journal of cheminformatics, 5(1), p.3.
45. Kass-Hout, T.A., Xu, Z., Mohebbi, M., Nelsen, H., Baker, A., Levine, J., Johanson, E. and Bright, R.A., 2016. OpenFDA: an innovative platform providing access to a wealth of FDA’s publicly available data. Journal of the American Medical Informatics Association, 23(3), pp.596-600.
46. Malone, J., Adamusiak, T., Holloway, E. and Parkinson, H., 2009. Developing an application ontology for annotation of experimental variables–Experimental Factor Ontology. Nature Precedings, pp.1-1.
47. Thompson, R.C., Simons, N.W., Wilkins, L., Cheng, E., Del Valle, D.M., Hoffman, G.E., Cervia, C., Fennessy, B., Mouskas, K., Francoeur, N.J. and Johnson, J.S., 2023. Molecular states during acute COVID-19 reveal distinct etiologies of long-term sequelae. *Nature medicine*, 29(1), pp.236-246.
